# Ovarian Dropsy

**Published:** 1849

**Authors:** Thompson Mead

**Affiliations:** Batavia Ill.


### ARTICLE II.

*Ovarian Dropsy.* By Thompson Mead, M. D., of Batavia Ill.

*March 11th* 1848.—Was called to Mrs. M. N., aged 33. She complained of an uneasy dragging sensation, occasionally pain in the left iliac region, and a feeling of weight and numbness in the corresponding hip; appetite variable, sometimes good and then poor; more or less nausea, with pain or distress in the stomach and coldness of the feet. The symptom she complained of as being the most constant, was the sensation of a fluid constantly falling, drop by drop, into the abdomen, which had increased with the accession of the other symptoms. Urine somewhat more scanty than in health, rather pale; bowels generally costive; the os uteri was situated higher than is usual. It was about a year since she first began to complain. By an external examination, I found the left side of the abdomen more prominent than the other, and deeply situated about midway between the ant. sup. spine of the ilium and the navel, a tumor as near as I could judge of the size of a fœtal head at six months. It was of considerable firmness, and somewhat firmly fixed in its situation, with no distinct evidence of any fluctuation. Of late, I was informed, it had increased in size quite fast. She could only lay on the left side. I was also informed that in the morning it was softer. As night approached the pain, sense of dragging, &c. increased.

Prescribed, in pill, blue mass, ext. hyosciam., and comp. ext. colocynth; iodine in solution of hyd. potash; a diuretic drink, and externally over the tumor, ung. iod. plumbi ʒj to ℥j lard.

Gradually her appetite improved, bowels became more regular, with less uneasiness about the tumor.

In June, after having felt remarkably well, she was attacked with pain in the region of the tumor, which was very severe, and from thence it extended over the whole abdomen with alternate chills and fever.

I saw her some three hours after this attack, which was sudden, and could discover no appreciable alteration of the tumor. Some 12 hours after I left, profuse diuresis came on. The next day I could not feel the least vestige of the tumor. I was then informed that as the diuresis continued the tumor diminished in size till it could not be felt, the tenderness of the abdomen gradually subsided. With the disappearance of the tumor a sensation of the fluid falling into the abdomen drop by drop also subsided. For a few weeks she continued to improve till she felt herself well, and resumed her labors about the house. The urinary discharge was described to me as being of a light color and without the ordinary smell of urine.

In the early part of Aug. there was a recurrence of the same symptoms, in the same order throughout the second attack.

*Sept. 3d.*—In the morning she was attacked with pain again in the tumor, and at night a profuse urinary discharge of the same character as that in the first attack, took place and with it a subsidence of the tumor and all the previous symptoms. She now, Nov., considers herself *well* and attends to her duties about the house.

